# Cleansing efficacy of an auto-cleaning device versus an oscillating- rotating toothbrush in home use. A pilot study in individuals with down syndrome

**DOI:** 10.1007/s00784-025-06203-w

**Published:** 2025-02-10

**Authors:** Dagmar Schnabl, Marwin Eller, David Trojer, Vera Wiesmueller, Franz Sebastian Schwindling, Ines Kapferer-Seebacher

**Affiliations:** 1 University Hospital for Dental Prosthetics, Medical University of Innsbruck, 6020 Innsbruck, Austria; 2 University Hospital for Conservative Dentistry and Periodontology, Medical University of Innsbruck, 6020 Innsbruck, Austria

**Keywords:** Trisomy 21, Disablement, Powered toothbrush, Automatic toothcleaning, Plaque index, Oral hygiene

## Abstract

**Objectives:**

People with intellectual disabilities often have poor oral hygiene and depend on carers’ support. We aimed to investigate, whether automatic toothbrushes could benefit people with Down syndrome (DS).

**Materials and methods:**

In a randomized, single-blinded cross-over study we compared the cleansing efficacy of a horse-shoe shaped automatic toothbrush with that of rotating-oscillating toothbrushing in unassisted domestic use over four weeks by persons with DS. Rustogi Modified Navy Plaque Index (RMNPI) and Gingival Bleeding Index (GBI) were assessed before and after each intervention period. Wilcoxon Signed-Rank Test was used for statistical analysis.

**Results:**

Fifteen participants (mean age 31 ± 8.33 years) finished the study. There were no statistically significant differences in RMNPI between the two brushing modalities after four weeks of unassisted home use, neither in full mouth (Y-brush®: median 59.2%; range 24.8 – 76.7; rotating-oscillating toothbrush: 54.6%; 6.4 – 71.3) (*p* = 0.484) nor in subgroup analyses. RMNPI was statistically significantly higher after four weeks of automated brushing than baseline. There was no statistically significant difference for full-mouth GBI between the two brushing modalities.

**Conclusions:**

Both, oscillating-rotating and automated toothbrushing resulted in unsatisfactory plaque control after unassisted use by people with DS.

**Clinical relevance:**

Further studies should investigate the impact of caregivers’ assistance with auto-cleaning devices to persons with disabilities on plaque removal efficacy. Customization of mouthpieces and simplification of handling modalities might effect a higher cleansing capacity and should be future goals for automatic brushing device manufacturers.

## Introduction

Down syndrome (DS) or trisomy 21 is the most frequent form of developmental intellectual delay caused by triplicate state of all or a critical portion of chromosome 21 [1]. Its main clinical features include mental impairment and characteristic facies, hypotonic musculature, congenital defects of the heart and gastrointestinal tract, neurobiological alterations, respiratory diseases, and a significantly higher risk of developing infection [1]. Characteristic oral features of individuals with DS are a mild mandibular prognathism and a hypoplastic maxilla, macroglossia, microdontia, short roots, tooth agenesis, delayed tooth eruption, dry mouth due to mouth breathing and lack of lip sealing [2]. A recent clinical study compared oral health characteristics of children and adolescents with DS to an age-matched control group, showing increased rates of gingival inflammation and a greater number of severe malocclusion whereas there is conflicting evidence on a decreased or increased caries prevalence [3, 4]. A significant association between DS and periodontitis with an odds ratio of 3.93 (95% CI 1.81–8.53) and significantly higher probing depths in individuals with DS as compared to controls was shown in a recent meta-analysis including eleven clinical studies [5]. The high prevalence and severity of periodontitis cannot simply be attributed to poor oral hygiene but is based on abnormalities in both the innate and adaptive immune systems, including mild to moderate T-cell lymphopenia, reduced antibody responses, and impairments in chemotaxis and neutrophils phagocytosis [2]. Due to intellectual disablement and an impaired motor function most individuals with DS largely depend on their caretakers’ support or supervision, also when it comes to domestic oral healthcare [6, 7].

The plaque reducing efficiency of toothbrushing, both with manual and electric toothbrushes, largely depends on the brusher’s understanding, motivation, and dexterity. In the general population, a small but statistically significant superiority of powered over manual toothbrushes was found for dental biofilm removal [8, 9], whereas two recent systematic reviews found no significant differences between manual and powered toothbrushing in people with physical or intellectual disabilities regarding plaque removal or gingival health [10, 11]. This applied to both self-brushing and caregiver brushing. Lately, automatic toothbrushes acquired vogue. Pre-eminently designed to accommodate neglectful toothbrushers by simple handling and a reduced brushing time through simultaneous cleaning of all teeth per jaw or mouth, automatic toothbrushing devices have so far not conclusively met expectations concerning plaque removal. A clinical study testing the auto-cleaning device Amabrush® (Vienna, Austria) assessed an insufficient fit of the horse-shoe shaped mouthpiece with diverse dental arches and an inappropriate bristle alignment resulting in poorer plaque removal as compared to uninstructed manual brushing in young healthy volunteers [12]. Another recent study compared the cleansing efficacy of the auto-cleaning device Y-brush® (Lyon, France) with that of manual toothbrushing in a single brushing exercise in 20 healthy probands [13]. Full-mouth plaque reduction was significantly lower with auto-cleaning for 5 s per jaw than with manual toothbrushing with a statistical significance in marginal and interdental sites but not in smooth tooth surfaces. Increasing the brushing time to 15 s per jaw resulted in a full-mouth plaque reduction comparable to manual toothbrushing (*p* = 0.177). Statie et al. [14] showed in a crossover randomized trial that the Y-brush (10 s per jaw) was significantly more effective than no brushing (negative control) but less effective than manual toothbrushing in dental plaque removal [15]. Authors of both studies concluded that the auto-cleaning device might be recommendable for individuals with low dexterity.

For individuals with intellectual disabilities and/or impaired motorfunction and a resulting poor plaque removal competence through conventional toothbrushing, an automatic toothbrushing device might constitute a beneficial tooth cleaning modality and promote self-reliance. Therefore, the aim of the present randomized and single-blinded cross-over study was to compare the cleansing efficacy of the auto-cleaning device Y-brush® with that of the oscillating-rotating toothbrush Oral-B® Pro 3 3000 in unassisted domestic use over a period of four weeks by persons with DS. The null hypothesis was that there would be no difference in cleansing efficacy between the two brushing modalities.

## Material and methods

The present study was conducted in accordance with the 1964 Declaration of Helsinki and its later amendments. Ethical approval was obtained by the Ethics Committee of the Medical University of Innsbruck, study number 1108/2022. The study was registered at the registry for clinical studies of the University Hospital of Innsbruck (Koordinationszentrum für klinische Studien; kks-innsbruck@i-med.ac.at), registration number 20221123–3057. Legal guardians of all participants and all participants themselves gave their informed written consent prior to the study enrollment.

### Subjects

Sample size calculation (please see *statistical analysis*) determined a sample size of nine. To increase the validity of this study, we aimed to include a sample of 20 individuals.

Inclusion criteria comprised 1) the diagnosis of DS, 2) minimum age of 18 years, 3) presence of at least ten teeth per jaw and of at least four teeth per quadrant, 4) community periodontal index of treatment needs (CPITN) [16] grade 1 or 2.

Exclusion criteria were 1) pregnancy or breastfeeding, 2) concurrent participation in another study, 3) presence of decayed teeth, and 4) presence of active orthodontic appliances. Teeth with direct or indirect restorations or dental implants were not excluded.

Recruitment of participants was carried out from December 2021 to November 2022 by means of a circular sent out by the Down Syndrome Association Tyrol (Verein Down-Syndrom Tirol).

### Clinical intervention

**Fig. 1:**
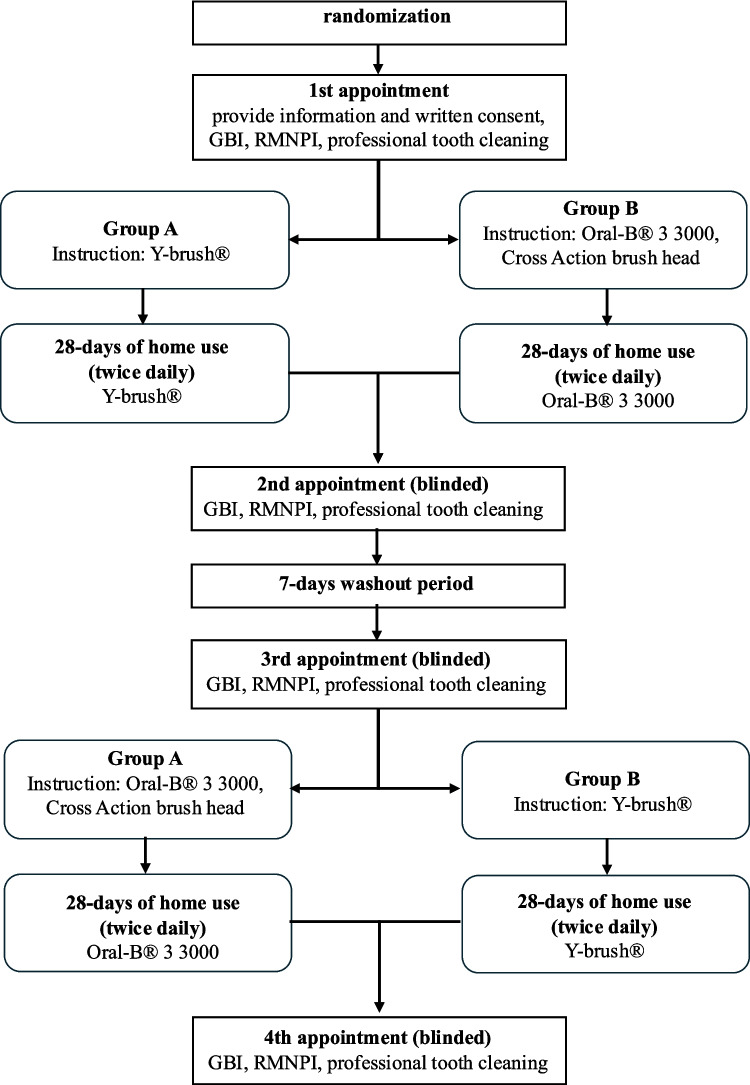
Visual outline of the study design

The cleansing efficacy of brushing with the auto-cleaning device Y-brush® versus a rotating-oscillating toothbrush was evaluated in a randomized, examiner-blinded, two-period crossover study. Each subject was invited to attend four appointments (Fig. 1).

At appointment one, each subject and his/her legal guardian were informed about the study procedure and signed an informed consent form after confirmation of in- and exclusion criteria. At baseline, the gingival bleeding index (GBI; Ainamo and Bay) was assessed at six sites per tooth [17]. After plaque disclosing by applying the solution (2Tone, Young, Earth City, Mo, USA) with a sponge pellet and rinsing thoroughly with water, the Rustogi Modified Navy Plaque Index (RMNPI) [18] was assessed by one blinded and trained examiner. Professional tooth cleaning was accomplished with sonic/ultrasound or manual scalers (in case of present calculus) and rubber cups with polishing paste (Cleanic®, Kerr, Bioggo, CH). Each participant was instructed in the use of the respective toothbrushing device that was allocated by computer-generated randomization, and provided with toothpaste (Sensodyne PRO Schmelz, GlaxoSmithKline Pharma GmbH, Vienna, Austria). Participants’ allocation to group 1 (“Y-brush® first”) or 2 (“Oral-B® Pro 3 3000 first”) was accomplished prior the study by Excel (Microsoft, Redmont, Washington, USA) with the random function *Zufallszahl.*

Twice daily use of the assigned brush and refraining from the adjunct use of any other toothbrush, toothpaste, any interdental cleaning device, mouthrinse, or chewing gum were commanded. Caretakers were instructed not to assist the brushing procedure.

The second appointment took place after four weeks’ use of the assigned toothbrush. Again, GBI and (after plaque disclosure) RMNPI were scored by the same blinded examiner. After professional tooth cleaning, a one-week wash-out phase was enrolled. Probands were told to resume their accustomed oral hygiene as before the study.

At the third appointment, GBI and RMNPI scores were taken by the same examiner and the handling of the other toothbrush to be tested was instructed. The fourth appointment included blinded GBI and RMNPI assessment and final professional cleaning.

### Y-brush®

**Fig. 2:**
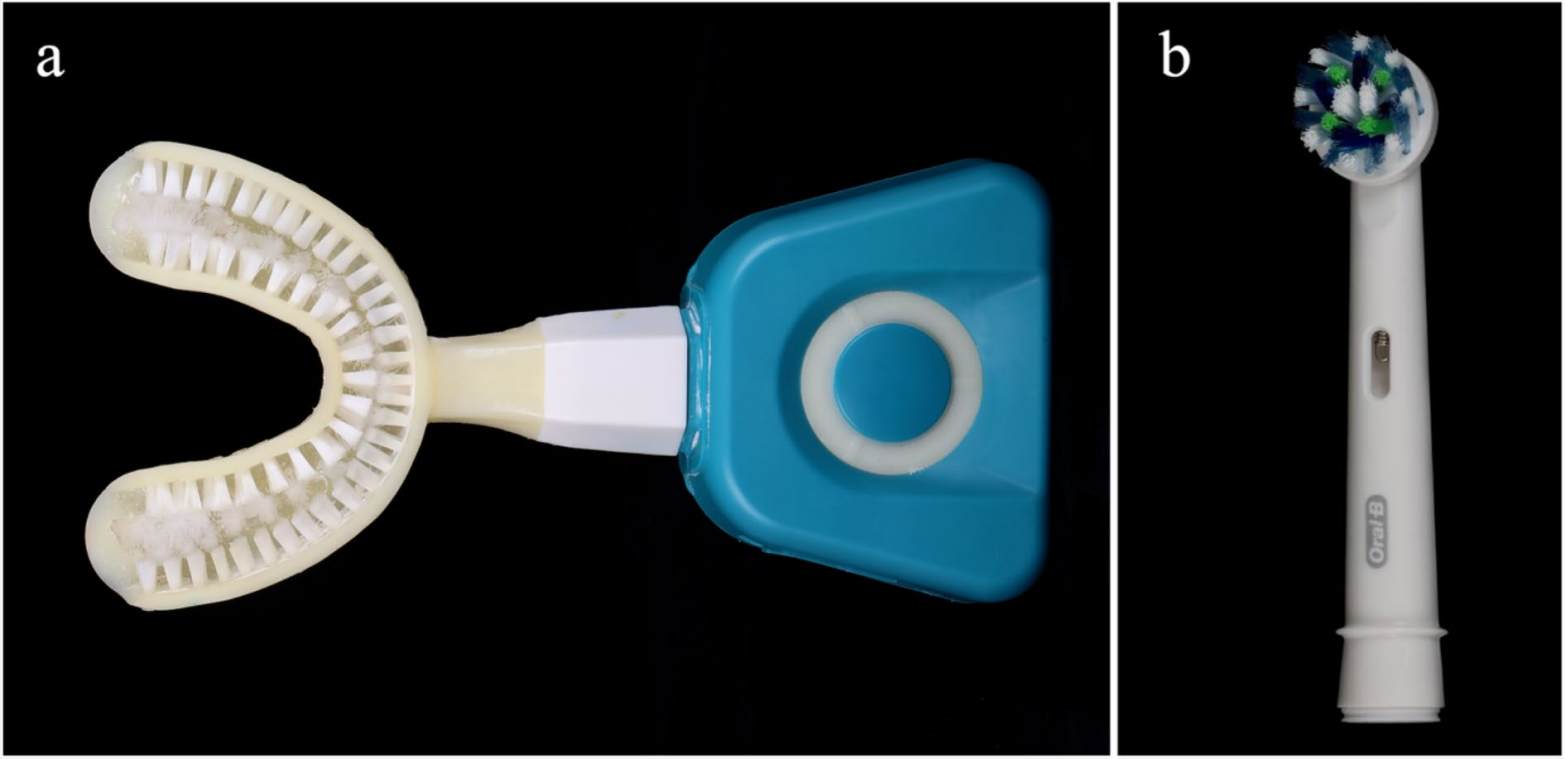
** a** The auto-cleaning device Y-Brush® has a horseshoe shaped flexible mouthpiece, mounted with nylon bristles on one side, which are inclined at a 45° angle to the gums. **b** The control product was the Oral-B® Cross Action brush head used with the oscillating-rotating toothbrush Oral-B® 3 3000

The horseshoe shaped flexible mouthpiece has been designed to clean all teeth of one jaw at the same time (Fig. 2A). It is on one side mounted with nylon bristles, which are inclined at a 45° angle to the gums to mimic the BASS technique. In this study, size M for adults, which is designated for age 12 plus, was used. Probands were instructed to wet the mouthpiece, load it with toothpaste by use of the supplied silicone toothpaste applicator, to insert and adjust it to the upper dental arch to ensure maximum fit, and to use the 15 s mode. After starting the cleaning process by pressing the start button, the mouthpiece should gently and quickly be chewed on and moved by the handle to the left and right side according to the manufacturer’s user manual [19]. The procedure was to be repeated in the lower jaw by turning the mouthpiece around with bristles and operating button facing downward and placing it onto the lower dental arch.

### Oral-B® pro 3 3000

The second toothbrush used in this study was the rotating-oscillating brush Oral-B® Pro 3 3000 with the brush head Oral-B® CrossAction (both Oral B, Procter & Gamble, Cincinnati, Ohio, USA) (Fig. 2B), which is mounted with about 2.200 bristles in a 16-degree angle. Probands were instructed to use the mode “daily cleansing”. They were briefed to brush systematically tooth by tooth occlusal sites first, oral sites second, and vestibular sites third in each jaw, placing the brush head at right angles onto the respective site and keeping it there for three seconds. Paying attention to the green light of the brush’s contact pressure control should ensure adherence to the correct pressure.

### Rustogi modified navy plaque index [18]

The index divides buccal and lingual surfaces into nine areas (A to I) that are scored for the presence (score = 1) or absence (score = 2) of plaque. It assesses the amount of plaque on a nine surface basis (areas A-I), smooth surface basis (areas E, G, H, and I), interdental surface basis (areas D and F), and the gingival margin surface basis (areas A-C). Third molars were excluded from the evaluation, whereas teeth with fillings, inlays, onlays, or crowns were included. RMNPI is calculated as percentage of biofilm adhering sites to measured sites.

### Gingival bleeding index (ainamo and bay) [17]

The gingival bleeding index (GBI) was determined by moving a periodontal probe within the gingival sulcus from the middle of the respective tooth towards the mesial and distal papilla. After 30 s, the presence or absence of bleeding was recorded in a dichotomous manner at six sites per tooth: mesio-buccal, buccal, disto-buccal, mesio-lingual, lingual, and disto-lingual. GBI was calculated as percentage of bleeding sites to evaluated sites.

### Statistical analysis

Sample size calculation was based on mean values and standard deviations of whole-mouth RMNPI scores provided by [13]. In that clinical investigation the cleansing efficacy of the Y-Brush® was compared with that of uninstructed manual toothbrushing in periodontally healthy individuals without disabilities in a single brushing exercise. Mean RMNPI after brushing was 13.04 ± 5.18% for manual toothbrushing and 29.8 ± 10.17% for the auto-cleaning device. Based on these data, sample size calculation for dependent samples, a power of 90% and α = 0.01 revealed a sample size of nine for the present investigation.

For descriptive analysis and if not stated otherwise, median and range are given.

On a participant-level, RMNPI values were calculated as the total number of tooth areas with plaque present divided by the total number of tooth areas scored. An analogous calculation of the GBI was performed. The main outcome of whole-mouth RMNPI as well as GBI scores were compared between the two toothbrushing procedures using the Wilcoxon signed-rank test. The statistical analysis was conducted using IBM SPSS Statistics V.29.0.0.0 (IBM Armonk; NY, USA. The significance level was *p* < 0.05, with a power of 80%.

## Results

### Subjects

Sixteen Caucasians with DS were recruited to participate in this study. One proband dropped out during the second intervention period, because she objected to the unaccustomed automatic cleansing protocol. Ten men and five women (mean age 31 ± 8.33 years) finished the study.

### Rustogi modified navy plaque index

**Fig. 3:**
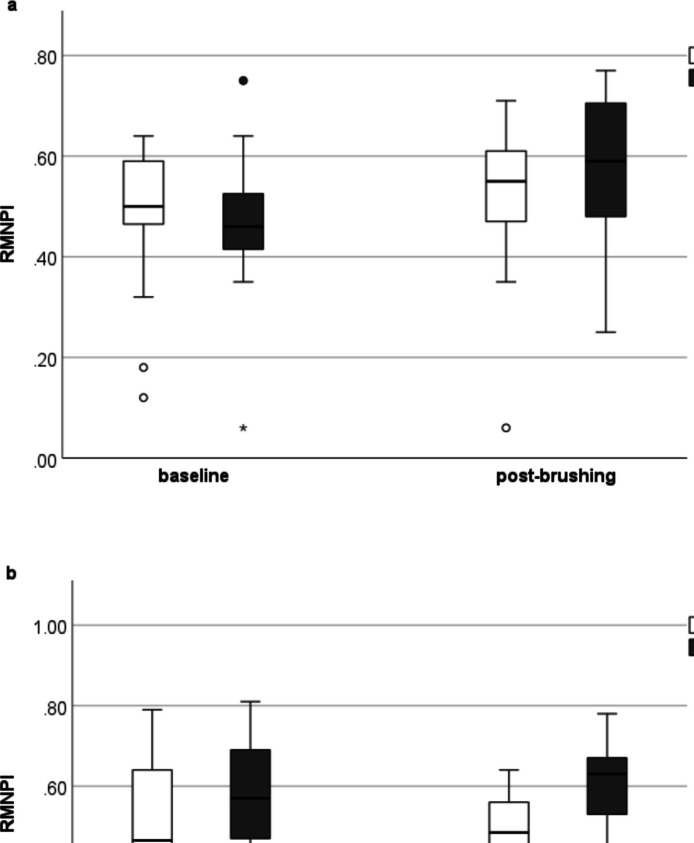
** a** Similar Rustogi Modified Navy Plaque Index (RMNPI) scores were detected at baseline and after four weeks of unassisted home use with both brushing modalities (*p *= 0.208, and *p* = 0.484, respectively). **b** RMNPI levels were lower in the upper jaw than in the lower jaw with both toothbrushes (Y-brush, *p* = 0.029, and rotating-oscillating brush, *p* = 0.004, respectively)

Baseline full-mouth RMNPI scores were not statistically significantly different between the two brushing modalities (Y-brush®: 46.2%; range 6.3 – 75.2; Oral-B® Pro 3 3000: 49.6%; 11.7 – 63.9) (*p* = 0.208). After four weeks of twice daily (unassisted) brushing there was no statistically significant difference between the two interventions (*p* = 0.484), although RMNPI scores increased to 59.2% (24.8 – 76.7) for brushing with the Y-brush® (*p* = 0.024) and to 54.6% (6.4 – 71.3) for brushing with the rotating-oscillating toothbrush (*p* = 0.342). There were no statistically significant differences in RMNPI subgroup analyses between the two groups (Table 1). For both brushing modalities, baseline and post-brushing RMNPI scores were statistically significantly lower in the upper than in the lower jaw (Fig. 3). For both brushing modalities, baseline and post-brushing RMNPI scores were statistically significantly lower in oral than in vestibular sites.

**Table 1:** Rustogi modified navy plaque index (RMNPI) baseline and after four weeks of unassisted home use

	Y-Brush®	Rotating-oscillating toothbrush	
Full mouth RMNPI	Median (range), %	Median (range), %	*p*-value
baseline	46.2 (6.3 – 75.2)	49.6 (11.7 – 63.9)	0.208
4-week brushing	59.2 (24.8 – 76.7)*	54.6 (6.4 – 71.3)	0.484
**Approximal surfaces**			
Baseline	59.0 (1.8 – 95.5)	59.0 (10.7 – 85.7)	0.582
4-week brushing	73.9 (32.1 – 91.1)	65.2 (6.5 – 91.3)	0.484
**Marginal surfaces**			
Baseline	75.6 (11.9 – 98.2)	75.7 (21.4 – 92.3)	0.503
4-week brushing	88.4 (30.4–100)*	85.3 (14.2 – 98.6)	0.704
**Smooth surfaces**			
Baseline	17.5 (4.5 – 47.8)	26.6 (4.9 –34.2)	0.226
4-week brushing	29.8 (13.0–54.3)*	25.0 (0.5 – 40.8)	0.187
**Upper jaw**			
Baseline	41.7 (10.3 – 78.6)	39.3 (2.6 – 61.1)	0.764
4-week brushing	55.6 (20.4 – 71.7)	49.1 (2.5 – 64.1)	0.453
**Lower jaw**			
Baseline	52.3 (2.4 – 71.8)	64.5 (28.5 – 81.0)	0.197
4-week brushing	64.5 (28.5 – 81.0)*	60.7 (9.0 – 77.8)	0.358

*p < 0.05

### Gingival bleeding index

**Fig. 4:**
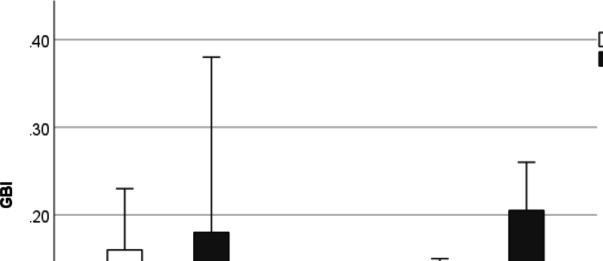
Similar gingival bleeding index (GBI) scores were detected at baseline and after four weeks of unassisted home use with both toothbrushes

Baseline GBI scores were not statistically significantly different between the two brushing modalities (Y-brush®: 5.1%; range 0.6 – 38.1; Oral-B® Pro 3 3000: 8.3%; 2.6 – 23.2) (*p* = 0.271). While there was a statistically significant decrease between baseline and post-intervention scores for the rotating-oscillating toothbrush (GBI 4.0% (0.0 – 15.5) (*p* = 0.027), there were no significant differences after brushing with the Y-brush (GBI 11.5%; 0.6 – 26.2) (*p* = 0.374), both showing a wide range (Fig. 4).

## Discussion

A limited ability to accomplish sufficient oral hygiene, along with other restrictions, results in poorer periodontal status conditions and higher unmet caries treatment needs in individuals with intellectual disabilities [20–22]. Improvement of oral care in disabled individuals is a general necessity to adjust oral health inadequacies [23]. [13] found a comparable plaque reduction of the auto-cleaning device Y-brush® to that of manual toothbrushing, when the brushing time of auto-cleaning was 15 s per jaw. They therefore hypothesized that auto-cleaning devices might be at least recommendable for people with low manual or cognitive skills, who would be expected to be less effective in plaque removal by self-use of manual or conventional powered toothbrushes [13]. Consequently, in the present study we compared the cleansing efficacy of the Y-brush® with that of an oscillating-rotating toothbrush (Oral-B® Pro 3 3000 and the cross-action brush-head) in unassisted domestic use by persons with DS.

The formulated null hypothesis was verified through this clinical cross-over investigation. Neither of the tested brushes prevailed over the other regarding post-brushing RMNPI in unassisted (twice daily) use by individuals with DS over four weeks. Moreover, in unassisted use both tested brushes compared poorly with customary daily oral hygiene routinely applied before the study and during the wash-out period (Table 1). Participants’ mode of toothbrushing before the onset of the study and during the wash-out period was not recorded in detail, but caretakers were to a varying extent involved in the cleansing procedure in the sense of supervision, assistance, or complete performance. Additionally, a bias cannot be ruled out, as participants and/or caretakers might have paid particular attention to oral hygiene before the study or during washout.

The current study was conducted as a pilot study. Although we aimed to include 20 participants, our study population consisted finally of 15 individuals aged 31 ± 8.33 years. A post hoc estimation of the power for P(X > Y) = 0.6 resulted in a power of 0.1559 (Wilcoxon signed rank test). A follow-up study with a power of 80% and a two-sided alpha of 0.5 would have to include 131 subjects. In the present study, we even had unsolvable problems recruiting the targeted 20 test subjects, despite personal contacts to the self-help group. A follow-up study would therefore probably have to be planned as a multicenter study or in a non-dental setting. Social cohesion within an association reflects team spirit and caretakers’ and participants’ willingness to collaborate on collective projects. Thus, a selection bias in the sense of recruitment of participants, who are supported by most diligent caretakers that pursue the best care for their dependents, is probable.

The findings of the study can also be interpreted to indicate that the automated toothbrush is equally effective as a rotating-oscillating toothbrushing, with the added benefit of requiring significantly less effort/time. Effective brushing with rotating-oscillating brushes, as commonly recommended and instructed in this study, requires adherence to a predefined sequence and a certain dexterity to place the brush correctly onto each tooth surface. Both intellectual and/or motor function impairment present in individuals with DS might be causes for poor plaque control by use of this kind of powered brush. But also the handling of the auto-cleaning device Y-brush® poses some difficulties, beginning with the loading with a specified amount of toothpaste by means of the provided silicone applicator, which is not firmly enough connectable to the toothpaste tube. According to instructors’ observations, the insertion and adjustment of the mouthpiece to the upper or lower jaw were frequently stymied by macroglossia. One and the same button serves to start the cleansing procedure (first press) and to select the duration of brushing cycle (second press). Operation of this button might have overstrained study participants and consistent use of the demanded 15 s mode is not assured. When – after turning the device from one jaw to the other – the button is faced downwards, its handling is additionally hampered with lacking vision control. During the cleansing process, the mouthpiece should be chewed on to facilitate a certain pressure and at the same time should be manually twisted by the handle to the left and right side in order to sufficiently cover posterior teeth, a procedure maybe too complex for disabled persons and probably not exerted correctly by study participants. The difficulties in handling of the Y-brush® could perhaps be compensated by supervision and/or assistance by caregivers. An increase of brushing time to e.g. 30 s per jaw might to some extent also offset delayed or inadequate auto-cleaning device operation. The aim of this study was to evaluate unassisted use of both tested brushes. It is not assured that either of the tested brushes was used correctly. Special anatomical conditions such as macroglossia and jaw incongruity [24, 25] might be the reason for less plaque accumulation in the upper than in the lower jaw and in oral than in vestibular tooth sites, which was found at baseline as well as at post-brushing assessments with both tested brushes.

Before release, participants were asked, which brush they prefer in daily use. Nine participants stated that they preferred the Oral-B® Pro 3 3000 brush over the Y-brush® and six participants favored the auto-cleaning device. The fact that the majority of participants preferred the Oral-B® Pro 3 3000 brush over the auto-cleaning device hints a certain objection to the after all intricate automatic cleaning procedure and a necessity to deskill commercial devices that are intended for the usage by/in persons with intellectual or physical disabilities.

The inaccurate fit of pre-manufactured auto-cleaning devices with diverse dental arches and its inadequate bristle alignment, analyzed in study participants’ plaster casts, have already been criticized by [13]. In this study, we refrained from impression taking and plaster cast analysis to keep participants’ strain down and to not compromise cooperation. In individuals with DS, malocclusion, namely class III malocclusion and anterior crossbite, are more prevalent than in the general population [25]. Intra-individual discrepancies of dental arch sizes might additionally limit the use of arbitrary mouthpieces. In very small (upper) jaws the M size mouthpiece may have been too large. Authors claim that individually (per jaw) customized mouthpieces (including tailored bristle alignment) based on dental models might be a promising future approach to enhance plaque removal efficacy of automatic toothbrushing devices. Three-dimensional digital intraoral scanning and the generation of printed or virtual models might facilitate the industrial production of customized mouthpieces, the technical feasibility and economic viability/costs of which are still to be calculated.

## Conclusion

In the present pilot study neither the auto-cleaning device Y-brush® nor rotating-oscillating toothbrushing reached satisfactory plaque levels in unassisted use by persons with DS. Further studies should investigate the impact of caregivers’ assistance with auto-cleaning devices to persons with disabilities on plaque removal efficacy.

## Data Availability

No datasets were generated or analysed during the current study.
